# Birt–Hogg–Dubé Syndrome: A Mini Review of the Clinical Manifestations, Investigation, and Management

**DOI:** 10.3390/jpm15120583

**Published:** 2025-12-01

**Authors:** Christina Ntinidi, Ioannis Tomos, Andreas M. Matthaiou, Nikoleta Bizymi, Adamantia Liapikou

**Affiliations:** 1 5th Department of Respiratory Medicine, Sotiria Thoracic Diseases General Hospital of Athens, 11527 Athens, Greece; christinadi296@gmail.com (C.N.); matthaiou.andreas@gmail.com (A.M.M.); nikoletabizymi@yahoo.gr (N.B.); mliapikou@yahoo.com (A.L.); 2 Laboratory of Molecular and Cellular Pneumonology, Medical School, University of Crete, 70013 Heraklion, Greece; 3 Respiratory Physiology Laboratory, Medical School, University of Cyprus, 2029 Nicosia, Cyprus; 4 Department of Nursing, School of Health Sciences, National and Kapodistrian University of Athens, 11527 Athens, Greece

**Keywords:** Birt–Hogg–Dubé syndrome, rare disease, genetic disease, cystic lung disease

## Abstract

Birt–Hogg–Dubé (BHD) syndrome is a rare genetic disease, inherited in an autosomal dominant manner, that was first described in the mid-1970s and occurs due to pathogenic variants in the folliculin gene (*FLCN*) on chromosome 17p11.2. The syndrome has numerous clinical manifestations and primarily affects the lungs, kidneys, and skin. As far as the pulmonary features are concerned, more than 80% of patients appear to develop bilateral pulmonary cysts located in the lower lung zones, in the subpleural area, with cumulative risk of spontaneous pneumothorax depending on the number of cysts in the lungs. Another serious feature of the syndrome is the increased risk of renal cell carcinoma, which is often an incidental finding on screening or medical imaging. Cutaneous manifestations include benign fibrofolliculomas, trichodiscomas, and acrochordons (skin tags), which primarily affect the patients’ emotional status as a result of their cosmetic defects. BHD syndrome is generally an underdiagnosed condition due to the great variability of its clinical picture, thus highlighting the importance of genetic testing for *FLCN* mutations in suspected cases. The application of ERN GENTURIS guidelines in clinical practice can facilitate early, accurate diagnosis of the disease and optimal personalized management of the patients.

## 1. Introduction

Birt–Hogg–Dubé (BHD) syndrome is an uncommon, autosomal dominant genetic disease associated with multisystemic manifestations, primarily cutaneous, pulmonary, and renal. The syndrome occurs due to pathogenic variants in the folliculin gene (*FLCN*), and it is related to the development of multiple pulmonary cysts with increased risk of spontaneous pneumothorax, distinctive skin lesions called fibrofolliculomas, and renal cell carcinoma (RCC) [1]. The first to describe the condition were Arthur Birt, Georgina Hogg, and William Dubé in the mid-1970s [2], and since then, BHD syndrome has been widely analyzed and described as a distinct genetic syndrome with multisystemic involvement. Early diagnosis is crucial due to the association of the syndrome with a high risk of RCC.

Although recent medical research has substantially enhanced the understanding of the pathophysiology and clinical features of the syndrome, BHD is still underdiagnosed, as it presents with variable clinical manifestations, the majority of which usually overlap with other medical conditions. Importantly, there might be a prolonged latent period between initial clinical manifestations and definitive diagnosis, leaving individuals at high risk without the appropriate oncological surveillance during this critical interval [3,4,5,6]. Importantly, BHD syndrome is one of the mammalian target of rapamycin (mTOR) pathway diseases, a group of rare genetic diseases sharing a common underlying molecular basis and providing the opportunity to combine basic and translational research as well as collaboration between basic scientists and clinicians [7]. The aim of this review is to summarize all recent data regarding the syndrome in order to provide clear and up-to-date diagnostic algorithms to ensure a more efficient personalized medical approach.

This is a narrative review, based on a critical, rather than systematic, evaluation of the literature. MEDLINE was searched through PubMed for recent articles with keywords that included, but were not limited to, terms such as “Birt-Hogg-Dubé”, “cystic disease”, “genetic”, “rare”, and “lung”. Importantly, publications regarding this rare genetic syndrome experienced a gradual increase after 2005, probably reflecting genetic innovations such as the introduction of next-generation sequencing (NGS) that year, which facilitated both research and clinical investigation and management. Further revolution in the field of genetics is reflected in the peak of publications regarding the syndrome after 2015, with more than 50 articles published on an annual basis. The authors focused, for the narrative synthesis of the review, on novel concepts and a personalized approach to diagnosis, treatment, and monitoring of BHD syndrome patients.

## 2. Epidemiology

The prevalence of BHD syndrome in the worldwide population is unclear. However, it is commonly estimated by studies at around 1 in 200,000 individuals, even though it is probably underestimated due to the underdiagnosis of the syndrome [8,9]. Notably, recent large-scale genomic studies have shown that pathogenic *FLCN* variants may appear a lot more frequently. Yngvadottir et al. have recently published their study from the United Kingdom (UK) using large-scale genomic registries (UK Biobank (UKB), 100,000 Genomes Project, and East London Genes & Health), analyzing exomes/genomes of 556,898 individuals and reporting the frequency of loss-of-function *FLCN* variants that are clinically validated as one in 4190 to one in 2710 [10]. There is no ethnic or gender predominance, yet the development of the syndrome appears to be age-dependent and typically manifests in the third or fourth decade of life with variable penetrance even among family members [11]. Exploring the family history is critical, as BHD syndrome may appear in several family members [12]. Importantly, age at onset of first symptom can be younger than expected in some individuals, and, thus, a thorough family history may prevent delayed diagnosis [11].

## 3. Genetic Pathogenesis and Molecular Basis of Disease

BHD syndrome results from mutations in the *FLCN* gene, which is located on chromosome 17p11.2. This gene encodes for folliculin, a tumor suppression protein involved in numerous signaling pathways. While the syndrome is inherited in an autosomal dominant manner, its pathogenesis follows the second-hit hypothesis. According to this model, patients are born with a germline mutation in one allele of the *FLCN* gene (“first hit”) and face a somatic mutation in the second allele in specific tissues during their life (“second hit”), leading to the loss of function in both alleles, the impaired production and/or function of folliculin, and tumorigenesis [3,4]. More than 150 different mutations in the *FLCN* gene have been identified, and around 50% of them are located in a polycytosine tract within exon 11 [3,4]. Of note, genetic testing and whole-exome sequencing have led to the continuous identification of novel mutations in additional exons in members of specific families, which predispose to malignancies, such as pulmonary adenocarcinoma [12,13]. In some cases, no detectable *FLCN* mutations can be found, while patients present a BHD syndrome-like phenotype. For instance, pathogenic variants of the *PRDM10* gene have been described, leading to impaired folliculin expression due to dampened affinity with the *FLCN* promoter in the absence of identifiable mutations in the *FLCN* gene itself [14].

Folliculin interacts with folliculin-interacting proteins 1 and 2 (FNIP1 and FNIP2), which are binding molecules that regulate the AMP-activated protein kinase (AMPK) and mTOR signaling pathways [3]. These pathways play a crucial role in cellular metabolism, cell growth, energy production, and autophagy. Inactivation or dysregulation of *FLCN* through the mutations described above leads to dysfunction of the AMPK and mTOR pathways, resulting in abnormal proliferation of the cells and oncogenesis, especially in renal tissues [15]. Additionally, another function of folliculin is to regulate lysosomal signaling, cellular adhesion via communication with proteins like plakophilin 4 and E-cadherin, and the transcription factors TFEB and TFE3 [15].

## 4. Clinical Features

BHD syndrome is described by manifestations in three organs: the lungs, the kidneys, and the skin. All the clinical manifestations might be present both independently of one another or in combination, which proves the diagnostic difficulty of the syndrome.

### 4.1. Pulmonary Manifestations

More than 80 of the BHD syndrome patients appear to develop pulmonary cysts, which are bilateral and mainly located in the lower lung zones, in the subpleural area [16,17]. One of the most common complications of the cysts is spontaneous pneumothorax, which is recorded to appear in around 24–38% of patients [18]. Of note, BHD syndrome is one of the most common causes of familial and recurrent pneumothorax [13,19,20]. The median age of the first occurrence of pneumothorax is approximately 38 years, although it has also occasionally been observed during childhood. Moreover, there is a cumulative risk of spontaneous pneumothorax associated with age and the number of pulmonary cysts, with a 75% recurrence rate with progressing age [21]. Despite the fact that BHD syndrome may appear with recurrent or large pneumothorax, pulmonary function is preserved in most cases. Interestingly, pneumothorax may even occur without cysts previously found on chest imaging. Consequently, BHD syndrome should be included in the differential diagnosis during the investigation of patients with recurrent spontaneous pneumothorax, cystic lung disease, or suggestive family history [21,22].

### 4.2. Renal Manifestations

Renal malignant tumors comprise the most serious clinical feature of BHD syndrome. Patients have an increased lifetime risk of developing RCC of 15–30%, which is seven times higher than in the general population. Renal tumors found in BHD syndrome are mostly bilateral and multifocal with a slow-growing rate [23]. Novel transcriptomic studies, involving NGS and both bulk and single-cell RNA sequencing, as well as immunohistochemistry, have shed more light onto the pathogenesis, pathology, and natural course of renal carcinogenesis in BHD syndrome [24,25,26,27]. The most common histological types described are hybrid oncocytic/chromophobe RCC, chromophobe RCC, oncocytoma, and occasionally clear cell RCC [23]. RCC occurrence is mainly recorded through midlife, although some cases have been reported to appear from the age of 20 years. RCC is usually an incidental finding on screening or medical imaging, and, despite its slow growth, metastases are common, thus making their early detection crucial for the prognosis of the disease. Prompt diagnosis justifies regular renal cancer surveillance for their affected family members, who have inherited the family’s *FLCN* mutation [28]. Of note, glycoprotein non-metastatic melanoma protein B (GPNMB) has been identified by Xia et al. as a diagnostic biomarker with high sensitivity for FLCN-mutated tumors, which could be utilized in the future to screen unclassifiable eosinophilic renal tumors [24].

### 4.3. Cutaneous Manifestations

**Figure 1:**
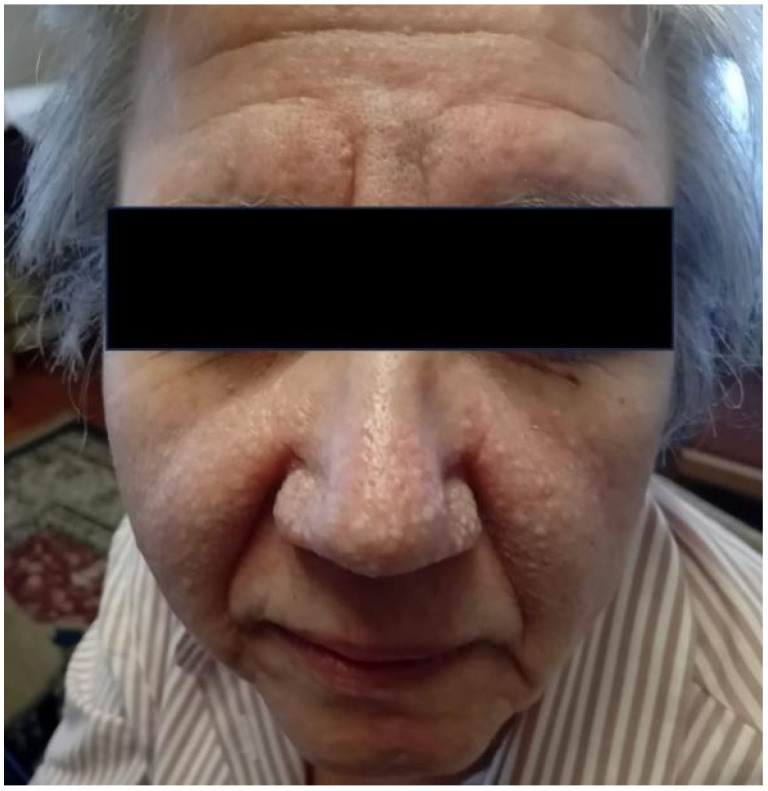
Photograph of a patient diagnosed with BHD syndrome showing the typical, small, papular cutaneous lesions over the facial region.

The most characteristic and specific skin manifestation of BHD syndrome is fibrofolliculoma, which is a benign tumor arising from the hair follicles (Figure 1). Fibrofolliculomas are more commonly described as multiple, dome-shaped papules of white color and are usually found on the face, neck, and upper trunk. These skin lesions typically occur after the age of 20 years during the third and fourth decades of life. Other skin lesions that are less common and less specific to the syndrome include trichodiscomas and acrochordons (skin tags). These skin lesions histologically appear to be proliferating epithelial strands that radiate from the hair follicles into a fibrous stroma, and, although benign, they often affect the patients’ emotional status for cosmetic reasons [2]. Unusual skin lesions have been described in the literature, such as atypical cutaneous fibrous histiocytomas, and may present in ultrarare cases of co-occurrence of germline pathogenic mutations in the *FLCN* gene and other gene variants, e.g., in *TP53*, which is implicated in Li-Fraumeni syndrome [29], further underlying the need for personalized surveillance.

However, broader manifestations may present with multiorgan involvement. While thyroid cancer is infrequent in BHD syndrome, thyroid nodules in these patients should raise suspicion [30,31]. The potential involvement of *FLCN* mutations in liver carcinogenesis and cholangiocarcinoma is still poorly understood, thus necessitating further research [32]. Moreover, whether hematologic malignancies, such as multiple myeloma, may be associated or symptomatic with BHD syndrome in patients with co-occurrence of the conditions is still debatable [33,34].

## 5. Clinical Investigation

Diagnosis can be made based on clinical features, imaging studies, histopathological findings, and/or genetic testing. Genetic testing for *FLCN* mutations confirms the diagnosis and allows cascade testing in families. Notably, a small percentage of patients with clinical BHD syndrome manifestations may not have detectable *FLCN* variants with conventional sequencing methods, while NGS provides a comprehensive molecular diagnosis by identifying exonic and intronic small nucleotide variants, small indels, and large intragenic deletions [35,36,37].

### 5.1. Diagnostic Criteria (European BHD Consortium)

According to the European BHD Consortium [35], the diagnosis of BHD syndrome is established when one major or two minor criteria are met, as follows:

- Major Criterion: >5 fibrofolliculomas/trichodiscomas with at least one confirmed histologically.
- Minor Criteria:
  1. Basally located bilateral pulmonary cysts with no other known cause.
  2. Early-onset (<50 years), multifocal, or bilateral renal cancer.
  3. First-degree relative with confirmed BHD syndrome.

According to ERN GENTURIS guidelines, the syndrome should be considered in individuals presenting with one or more of the following: characteristic cutaneous lesions, such as multiple fibrofolliculomas or trichodiscomas, renal manifestations, including RCC, and pulmonary cysts [38]. Specific clinical features that may raise suspicion of the syndrome include primary spontaneous pneumothorax, multiple bilateral pulmonary cysts (predominantly in the lower lung zones), bilateral or multifocal renal tumors (e.g., RCC and/or oncocytomas), RCC diagnosed before the age of 50 years or with a familial pattern, and clusters of skin papules consistent with fibrofolliculoma or trichodiscoma. However, clinicians should be aware that the syndrome also displays variable expression [35,38].

### 5.2. Differential Diagnosis

BHD syndrome may resemble several other medical conditions, requiring a wide differential diagnosis and thorough investigation:

- Emphysema: apical pulmonary cysts in tobacco smokers.
- Tuberous sclerosis complex (TSC): facial angiofibromas, renal angiomyolipomas, and cortical tubers.
- Lymphangioleiomyomatosis (LAM): diffuse pulmonary cysts in females, associated with TSC or sporadic.
- Langerhans cell histiocytosis (LCH): nodulocystic pulmonary disease in young adult smokers.
- Marfan and Ehlers-Danlos syndromes and other related genetically inherited conditions: pneumothorax and connective tissue abnormalities [35,38].

### 5.3. Genetic Testing

Genetic testing for *FLCN* to diagnose BHD syndrome should be performed in the presence of any of the following:

- Recurrent primary spontaneous pneumothorax and/or familial cases.
- Multiple bilateral pulmonary cysts, particularly in the lower lung zones.
- Bilateral or multifocal renal neoplasia.
- Familial or early onset (less than 45 years of age) RCC.
- Multiple cutaneous papules clinically consistent with fibrofolliculoma/trichodiscoma with at least one histologically confirmed fibrofolliculoma.
- Any combination of the aforementioned cutaneous, pulmonary, and renal manifestations in the same individual or family members [35,38].

### 5.4. Radiological Features

**Figure 2:**
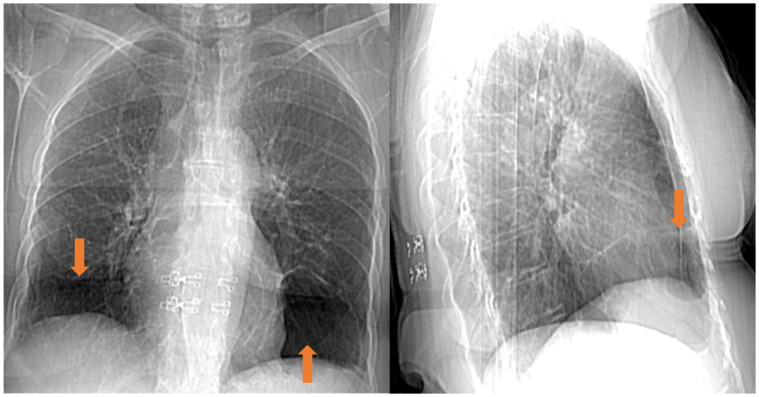
Chest radiograph of a patient with BHD syndrome showing thin-walled cysts (orange arrows), predominantly located in the lower lobes of the lungs.

**Figure 3:**
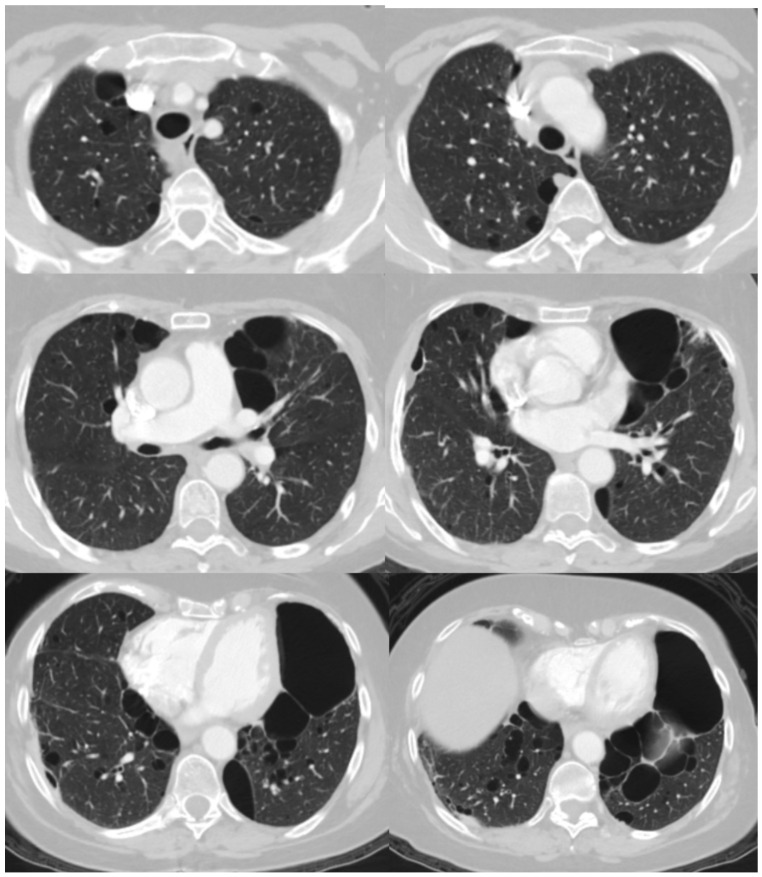
High-resolution chest computed tomography of a patient with BHD syndrome showing multiple, thin-walled pulmonary cysts with lower lung lobe predominance.

High-resolution computed tomography (HRCT) of the chest remains the gold standard method to depict the characteristic pulmonary lesions of the syndrome. Chest HRCT reveals small, thin-walled, lentiform cysts, predominantly located in the subpleural area of the lower lobes of the lungs (Figure 2 and Figure 3) [39]. This imaging technique can differentiate BHD syndrome from other diffuse cystic lung diseases (DCLDs), such as LAM, which is characterized by diffuse and uniform cysts, emphysema, which is more centrilobular and typically found mainly in the upper lobes, and LCH [40]. On the other hand, renal imaging is preferably conducted via magnetic resonance imaging (MRI) with contrast. In MRI scanning, renal tumors are typically described as enhancing solid masses without fat and are usually multifocal and bilateral [35,38].

### 5.5. Histopathological Features

Histologically, pulmonary cyst walls in BHD syndrome are lined with type I and II pneumocytes, surrounded by normal lung parenchyma, and appear to have no inflammation or oncogenic proliferation. Renal tumors show a variety of histological characteristics and are commonly described as hybrid oncocytic tumors with features of chromophobe RCC and oncocytoma. Fibrofolliculomas, the most common skin lesion of BHD syndrome, appear as small, dome-shaped cords of epithelial cells radiating from hair follicles and surrounded by fibrous stroma on the face, neck, and upper torso. Other skin lesions include trichodiscomas, acrochordons, and angiofibromas, typically occurring in adulthood and increasing with progressing age [35,38].

## 6. Clinical Management

Based on the fact that BHD syndrome has multiorgan manifestations, clinical management of BHD syndrome patients requires a multidisciplinary team approach, including specialists from pulmonology, nephrology, dermatology, thoracic surgery, and urology, in collaboration with radiology, medical oncology, and medical genetics [41]. Multicenter collaborative studies can provide support to clinicians with limited experience in BHD syndrome due to its rarity. Such a study was conducted by Namba et al. in Japan, including 155 patients from 33 hospitals, which were provided expert support by the referral center of the country, proving that such support programs facilitate the standardization of clinical practice of rare conditions at a nationwide level [42]. Moreover, initiatives such as the mTOR Pathway Diseases node, part of the Rare Disease Research UK platform, enable translational research and improvement in understanding of the pathogenesis and management of rare conditions, including BHD syndrome [7].

A baseline chest HRCT is highly recommended at the diagnosis of BHD syndrome to depict the extent and severity of pulmonary manifestations. Patients should be educated about the fact that pneumothorax can be a life-threatening complication when engaging in activities that involve rapid changes in atmospheric pressure, such as high-altitude activities or scuba diving, and that it is important to quit smoking [43]. If the first spontaneous pneumothorax occurs, patients can undergo thoracic tube placement and pleurodesis to minimize recurrence risk. However, there is still a high rate of recurrence. Several studies underscore the use of video-assisted thoracoscopic surgery (VATS) followed by talc pleurodesis or partial pleurectomy. Cases of patients who even needed VATS to be reperformed have been described [13,19,20,44,45]. It is also recommended that BHD syndrome patients undergo pulmonary function testing regularly [35,38]. Personalized approach, including pulmonary rehabilitation, assessment of exercise limitation and fitness to work, especially in occupations requiring the ability to exercise, such as military positions, is of great importance [19,46].

BHD syndrome patients should undergo renal surveillance through abdominal MRI with intravenous contrast for lifelong RCC monitoring, considering its high sensitivity and safety. Abdominal MRI is generally preferred over ultrasonography, which, however, can be used if MRI is not available. Renal imaging is recommended to begin as soon as the diagnosis is made and repeated every 1–3 years, depending on the MRI findings. Renal tumors that are smaller than 3 cm in size are recommended to be monitored yearly, while larger lesions require nephron-sparing surgery or other techniques, such as partial nephrectomy, which is minimally invasive and avoids intraoperative blood loss, so that renal function is preserved [47]. Less invasive techniques, such as cryoablation and radiofrequency ablation, are mainly available for patients who are not eligible for surgery. The reason these techniques are not as recommended as the standard surgical approach is that, in case of a renal tumor recurrence, a previous ablation may complicate imaging, and, due to scar tissue development, it can also increase the difficulty of a future surgery [47]. Importantly, innovative and individualized approaches, such as targeted immunotherapy [48] and renal transplantation, appear in the literature as additional options in patients with RCC [49].

In patients with BHD syndrome undergoing surgery, a special emphasis should be given to tailored and carefully designed anesthesia, as far as the sedative medications, lung-protective ventilation, and controlled extubation procedures are concerned, to the patients’ needs in order to minimize the risks, including pneumothorax and renal function deterioration [50,51].

Moreover, surveillance for thyroid cancer, salivary cancer, and melanoma is not recommended to be included in the regular follow-up of the patients [35,38], as these types of malignancies have only been described as occasional cases among BHD patients [30,52]. On the other hand, management of skin manifestations in BHD syndrome is performed on an elective and personalized basis and is cosmetically oriented. Laser ablation, surgical excision, or electrosurgery comprise acceptable treatment modalities. Recurrence is quite often, and no pharmacotherapy, such as rapamycin, has been proven to be effective [35,38]. Strategies offering a combination of techniques, such as the one described by Lodi et al. using both fractionated ablative CO_2_ laser and a flash lamp pulsed dye laser, have been proposed as a means to reduce dermal residual lesion relapses through deeper ablation as well as to improve control over tissue healing [53].

Finally, as in any case of a suspected monogenic pulmonary disease, and especially because of the higher risk of several types of malignancies, genetic counselling constitutes an important aspect in the management of BHD syndrome. Adult first-degree relatives of BHD syndrome patients, as well as individuals with a strong clinical suspicion of the condition, are strongly recommended to undergo genetic screening to establish the diagnosis and initiate management early in the course of the disease [11,41].

## 7. Conclusions

BHD syndrome is a complex, underrecognized, genetic disease that appears with diverse clinical manifestations, mainly affecting the lungs, kidneys, and skin. Although most features of the syndrome are benign, RCC and spontaneous pneumothorax can be life-threatening. Thus, it is highly recommended that genetic testing be applied for early diagnosis. Growing awareness among clinicians can enhance the diagnostic rate of BHD syndrome, while further research is required to better understand the molecular basis of the condition, which could be promising for the development of personalized therapeutic approaches.

## Data Availability

The original contributions presented in this study are included in the article. Further inquiries can be directed to the corresponding author.
